# Altered Expression of Cell Cycle Regulators and Factors Released by Aged Cells in Skeletal Muscle of Patients with Bone Fragility: A Pilot Study on the Potential Role of SIRT1 in Muscle Atrophy

**DOI:** 10.3390/biomedicines13061350

**Published:** 2025-05-31

**Authors:** Angela Falvino, Roberto Bonanni, Beatrice Gasperini, Ida Cariati, Angela Chiavoghilefu, Amarildo Smakaj, Virginia Veronica Visconti, Annalisa Botta, Riccardo Iundusi, Elena Gasbarra, Virginia Tancredi, Umberto Tarantino

**Affiliations:** 1 Department of Biomedicine and Prevention, “Tor Vergata” University of Rome, 00133 Rome, Italy; angelafalvino95@gmail.com (A.F.); beatrice.gasp95@gmail.com (B.G.); amarildo.smakaj@gmail.com (A.S.); virginia.veronica.visconti@uniroma2.it (V.V.V.); botta@med.uniroma2.it (A.B.); 2 Department of Applied Clinical and Biotechnological Sciences, University of L’Aquila, 67100 L’Aquila, Italy; roberto.bonanni@univaq.it; 3 Department of Systems Medicine, “Tor Vergata” University of Rome, 00133 Rome, Italy; tancredi@uniroma2.it; 4 Department of Orthopaedics and Traumatology, “Policlinico Tor Vergata” Foundation, 00133 Rome, Italyriccardo.iundusi@uniroma2.it (R.I.); gasbarra@med.uniroma2.it (E.G.); 5 Centre of Space Bio-Medicine, “Tor Vergata” University of Rome, 00133 Rome, Italy; 6 Catholic University “Our Lady of Good Counsel”, 1026 Tirana, Albania; umberto.tarantino@uniroma2.it

**Keywords:** cellular aging, senescence, physiology, SIRT1, muscle atrophy, bone fragility, osteoporosis, fragility fractures

## Abstract

**Background/Objectives**: Cellular aging represents a crucial element in the progression of musculoskeletal diseases, contributing to muscle atrophy, functional decline, and alterations in bone turnover, which promote fragility fractures. However, knowledge about expression patterns of factors potentially involved in aging and senescence at the tissue level remains limited. Our pilot study aimed to characterize the expression profile of cell cycle regulators, factors released by aged cells, and sirtuin 1 (SIRT1) in the muscle tissue of 26 elderly patients undergoing hip arthroplasty, including 13 with low-energy fracture and 13 with osteoarthritis (OA). **Methods**: The mRNA expression levels of cyclin-dependent kinase inhibitor 1A (*CDKN1A*), cyclin-dependent kinase inhibitor 1B (*CDKN1B*), cyclin-dependent kinase inhibitor 2A (*CDKN2A*), *p53*, tumor necrosis factor alpha (*TNF-α*), interleukin-1 beta *(IL-1β*), interleukin-6 (*IL-6*), interleukin-15 (*IL-15*), chemokine (C-C motif) ligand 2 (*CCL2*), chemokine (C-C motif) ligand 3 (*CCL3*), growth differentiation factor 15 (*GDF15*), and *SIRT1* were evaluated in muscle tissue by qRT-PCR. In addition, immunohistochemistry and Western blotting analysis were conducted to measure the protein levels of SIRT1. **Results**: A marked muscle atrophy was observed in fractured patients compared to the OA group, in association with an up-regulation of cell cycle regulators and factors released by the aged cells. The expression of matrix metallopeptidase 3 (*MMP3*), plasminogen activator inhibitor 1 (*PAI-1*), and fas cell surface death receptor (*FAS*) was also investigated, although no significant differences were observed between the two experimental groups. Notably, SIRT1 expression was significantly higher in OA patients, confirming its role in maintaining muscle health during aging. **Conclusions**: Further studies will be needed to clarify the role of SIRT1 in the senescence characteristic of age-related musculoskeletal disorders, counteracting the muscle atrophy that predisposes to fragility fractures.

## 1. Introduction

The increase in the elderly population has intensified scientific interest in the biological and pathophysiological processes associated with aging, with the goal of developing therapeutic and preventive strategies to counteract and slow age-related changes [1,2]. Among these, cellular aging leading to senescence plays a central role, representing a biological mechanism closely related to the response to cellular stresses, including DNA damage, metabolic dysfunction, and chronic inflammation [3,4]. Aged cells, also known as senescent cells, which lose the ability to proliferate, are responsible for the release of specific factors, called senescence-associated secretory phenotype (SASP), consisting of cytokines, chemokines, proteases, and growth factors [5,6]. These are altered in various disease contexts, such as type II diabetes mellitus, atherosclerosis, and neurodegenerative diseases, as well as in age-related musculoskeletal disorders, impairing the structural and functional architecture of tissues and predisposing to the risk of disability and mortality [7,8,9,10].

In this context, muscle atrophy emerges as one of the most insidious age-related musculoskeletal changes, characterized by progressive deterioration of muscle mass, strength, and function and closely associated with altered release of SASPs by aged cells [11]. Impaired muscle function and structure has direct repercussions on bone tissue, contributing to the deterioration of its quality and predisposing to the onset of skeletal disorders, such as osteoporosis and subsequent fragility fractures [12]. Indeed, several evidences have been reported on the mutual influence between muscle and bone tissue, as reduced muscle mass and strength promote the onset of osteoporosis and poor bone architecture increases muscle impairment in aging [13]. However, although the release of SASPs by skeletal muscle may vary considerably in individuals with diseases that alter bone homeostasis, the pattern of muscle expression of these molecules in such patients remains poorly characterized [14].

Importantly, the accumulation of cell cycle inhibitors, such as cyclin-dependent kinase inhibitor 1A (*CDKN1A*)/p21, cyclin-dependent kinase inhibitor 1B (*CDKN1B*)/p27, and cyclin-dependent kinase inhibitor 2A (*CDKN2A*)/p16 could play a key role in the induction of replicative senescence and progression of age-related muscle atrophy [15,16]. p53 could also negatively regulate the cell cycle by participating in the muscle atrophy that characterizes aging, as a wide variety of conditions that produce atrophy, including immobilization, would appear to increase the expression of this cell cycle regulator [17]. However, it remains to be elucidated whether the concomitant presence of diseases that impair bone tissue, such as osteoporosis, may influence the muscle expression of these markers. Undoubtedly, targeting these factors could be a promising approach for the development of new therapeutic strategies to counteract age-related muscle atrophy and prevent associated bone tissue deterioration.

Among the SASPs that contribute to musculoskeletal decline, interleukin-1 beta (IL-1β) and tumor necrosis factor alpha (TNF-α) are those most involved in muscle aging, although numerous other molecules may be released from senescent cells and act as SASPs [14]. In particular, interleukin-6 (IL-6) has been suggested as a key regulator of muscle metabolism, with both pro- and anti-inflammatory effects. Indeed, while IL-6 release in response to exercise appears to promote muscle anabolism and reduce circulating TNF-α levels, in the context of sarcopenia, a chronic low-grade inflammatory environment is established in which IL-6 exerts pro-inflammatory effects and promotes muscle catabolism [18]. Furthermore, mRNA levels of interleukin-15 (*IL-15*) are known to increase significantly with age and under limb unloading conditions in experimental models, suggesting its involvement in the muscle atrophy and cellular senescence characterizing sarcopenia [19]. Similarly, increased expression levels of chemokine (C-C motif) ligand 2 (CCL2) were detected in C2C12 myoblasts with senescence induced by oleic acid treatment [20], whereas increased expression of matrix metallopeptidase 3 (MMP3) was detected in muscle fibers overexpressing p16 and p21 from mice with sarcopenia induced by 1,25-dihydroxycholecalciferol deficiency [21]. In addition, differentiation factor 15 (GDF15), chemokine (C-C motif) ligand 3 (CCL3), fas cell surface death receptor (FAS), and plasminogen activator inhibitor 1 (PAI-1) were indicated as SASP factors that could significantly influence musculoskeletal metabolism and play an important role in the progression of sarcopenia [22].

In this context, sirtuin 1 (SIRT1), an nicotinamide adenine dinucleotide (NAD)^+^-dependent protein deacetylase, emerges as a crucial regulator of muscle homeostasis, being involved in several essential cellular processes, including modulation of inflammation, DNA repair, regulation of energy metabolism, and inhibition of cellular senescence [23,24]. Therefore, a deeper understanding of the role of SIRT1 could offer new perspectives to effectively intervene on muscle atrophy and skeletal disorders associated with aging [25]. In particular, a deficiency of SIRT1 could negatively affect the expression of cell cycle inhibitors, promoting muscle cell aging and the subsequent release of pro-inflammatory factors. However, the differences in expression of SIRT1, cell cycle regulators, and factors released by aged cells in skeletal muscle tissue of elderly patients with osteoporosis and fragility fracture have not yet been elucidated.

Based on this evidence, our study aimed to (i) provide a characterization of the expression of mainly cell cycle regulators and relevant factors released by aged cells in the skeletal muscle of elderly patients with osteoporotic bone fracture or patients with osteoarthritis, and (ii) evaluate the expression of SIRT1 to suggest its potential involvement in cellular aging processes that lead to skeletal muscle senescence during aging.

## 2. Materials and Methods

### 2.1. Study Cohort

The study included 26 patients admitted to the Department of Orthopaedics and Traumatology of the Policlinico “Tor Vergata” Foundation. The enrolled subjects were divided into two experimental groups: 13 patients undergoing hip arthroplasty for fragility fracture (fractured) and 13 patients without fracture undergoing hip arthroplasty for coxarthrosis (OA).

Patients with endocrine disorders affecting bone metabolism, myopathies, neuromuscular diseases, cancers, chronic viral infections, diabetes, or a history of long-term corticosteroid use for autoimmune conditions or previous orthopedic surgical implants were excluded from the study.

### 2.2. Clinical Parameters

Bone mineral density (BMD) was assessed by dual-energy X-ray absorptiometry (DXA) using a Lunar DXA system (GE Healthcare, Madison, WI, USA). The lumbar spine (L1–L4) and femoral regions (neck and total) were scanned following the manufacturer’s guidelines [26]. BMD was reported in grams per square centimeter, with a coefficient of variation of 0.7%. For fractured patients, measurements were taken on the unaffected limb; while for all other participants, BMD was measured on the non-dominant side. During the procedure, patients lay supine on an examination table with their limbs slightly abducted. DXA scans were conducted one day prior to surgery for OA patients and 1-month post-surgery for fractured patients. Results were expressed as *T*-scores.

### 2.3. Sample Collection

Vastus lateralis muscle biopsies were collected during hip arthroplasty surgeries from each patient. A portion of the samples was stored at −80 °C for subsequent RNA extraction, while the remainder was used for morphometric, immunohistochemistry, and Western blotting analyses.

Sample processing was conducted according to the appropriate guidelines. All experimental procedures were approved by the Territorial Ethics Committee (CET) of Lazio Area 2 (approval reference number #25/23) and were performed according to the World Medical Association’s Code of Ethics (Declaration of Helsinki). Before surgery, all participants provided written informed consent.

### 2.4. Morphometric Analysis

Muscle biopsies were fixed in 4% paraformaldehyde for 24 h and subsequently embedded in paraffin. Tissue sections with a thickness of 3 μm were stained with hematoxylin and eosin (H&E) (Bio-Optica, Milan, Italy) for histological analysis. Images were captured at 40× magnification using a Nikon upright microscope ECLIPSE Ci-S (Nikon Corporation, Tokyo, Japan) equipped with a Nikon digital camera and NIS-Elements software (version 5.30.01; Laboratory Imaging, Prague, Czech Republic).

Morphometric analysis was conducted by two independent, blind observers, measuring the diameter of muscle fibers. Measurements were taken at 40× magnification, collecting a total of three nonoverlapping readings for each participant. A reference area was plotted using NIS-Elements software to keep the size of the region of interest constant in each measurement and to ensure the consistency of the assessments.

### 2.5. Immunohistochemical Analysis

Immunohistochemistry was performed on muscle tissue of all patients to explore the expression of SIRT1. Briefly, 3 μm-thick sections were pretreated with ethylenediaminetetraacetic acid (EDTA) citrate, pH 6.0 for 20 min at 95 °C and then incubated with mouse monoclonal anti-SIRT1 [19A7AB4] (dilution 1:100; ab110304, AbCam, Cambridge, UK) for 1 h at room temperature. Washings were performed using phosphate-buffered saline (PBS) with Tween-20, pH 7.6 (UCS Diagnostic, Rome, Italy). Antibody binding was visualized using a horseradish peroxidase (HRP)-3,3’-diaminobenzidine (DAB) detection kit (UCS Diagnostic, Rome, Italy). The immunostaining background was evaluated with negative controls for each reaction, incubating the sections with secondary antibodies only (HRP) or with the detection system only (DAB) (Figure S1A,B).

The expression of SIRT1 was measured by mean optical density (MOD), a semiquantitative methodology that assesses chromogenic signal intensity as an indicator of protein amount in tissue sections [27]. Image analysis was performed using NIS-Elements software (version 5.30.01; Laboratory Imaging, Prague, Czech Republic), which identified areas of interest containing the signal. MOD was calculated by determining the signal intensity within these areas, providing a relative estimate of protein concentration for each sample. For each condition, the experiment was conducted in triplicate (*n* = 15 from N = 5 experiments).

### 2.6. Western Blotting Analysis

SIRT1 expression levels were analyzed in muscle biopsies obtained from all patients by Western blotting analysis. Proteins were extracted from muscle tissues using RIPA buffer and separated under reducing conditions on 8–16% precast SDS-PAGE gels (Bio-Rad, Hercules, CA, USA). Protein concentrations were measured with the Pierce BCA Protein Assay Kit (Thermo Scientific, Vilnius, Lithuania). Equal amounts of protein (20 μg) were loaded onto the gels, separated by electrophoresis, and transferred to PVDF membranes, which were incubated with mouse monoclonal anti-SIRT1 antibody (ab110304, AbCam, Cambridge, UK). Secondary detection was performed with HRP-conjugated anti-mouse IgG. β-actin expression was used as a loading control by probing the same membranes with mouse monoclonal anti-β-actin-Peroxidase antibody (A3854, Sigma-Aldrich, St. Louis, MO, USA). Immunoreactive bands were visualized using enhanced chemiluminescence (ECL Advance, Amersham; GE Healthcare Life Sciences, Little Chalfont, Buckinghamshire, UK) and detected with a VersaDoc 5000 Imager (Bio-Rad).

The quantification of SIRT1 expression was performed by densitometric analysis of the bands using ImageJ software (NIH), normalized to β-actin expression, expressing as mean ± standard error. The original Western blotting images are shown in Figure S1C,D.

### 2.7. RNA Extraction and Real-Time Quantitative Polymerase Chain Reaction (qRT-PCR) Analysis

RNA extraction was performed using approximately 80 mg of tissue, which had been stored at −80 °C. In detail, 1 mL of TRIzol reagent and a stainless-steel bead were added to each tissue to facilitate the homogenization process. The tube was sealed with parafilm and homogenized with a homogenizer set at 25 Hz for 5–7 min. Then, the bead was removed with sterilized tweezers and the homogenized sample was centrifuged at 12,000× *g* for 10 min at 4 °C. The supernatant was carefully transferred to a new 2 mL tube, and the pellet was discarded. To ensure complete dissociation of the nucleoprotein complexes, the sample was left at room temperature for 5 min. Next, 200 µL of chloroform per mL of TRIzol was added and the sample was shaken for 15 sec before being left at room temperature for 2 to 5 min. Phase separation was achieved by centrifugation at 12,000× *g* for 15 min at 4 °C. The upper aqueous phase was collected and mixed with isopropanol, followed by centrifugation at 12,000× *g* for 10 min at 4 °C. The resulting RNA pellet was washed with 75% ethanol and air-dried for approximately 30 min. The purified RNA was then dissolved in 30 µL of RNase-free water and stored at −80 °C.

Approximately 500 ng of purified RNA, quantified with a NanoDrop ND-1000 Spectrophotometer (DeNovix Inc., Wilmington, DE, USA), was used for reverse transcription with the High-Capacity cDNA Reverse Transcription Kit (Thermo Fisher Scientific, Waltham, MA, USA). Real-time PCR was carried out using the Applied Biosystems^®^ 7500 Fast Real-Time PCR System (Life Technologies, Carlsbad, CA, USA). qPCR analysis was performed with the Power SYBR Green PCR Master Mix (Thermo Fisher Scientific, Waltham, MA, USA) following the following cycling conditions: 95 °C for 10 min, followed by 40 cycles of 95 °C for 15 sec and 58 °C for 1 min. Primer sequences are listed in Table 1. The relative gene expression levels of *CDKN1A*, *CDKN1B*, *CDKN2A*, *p53*, *TNF-α*, *IL-1β*, *IL-6*, *IL-15*, *CCL2*, *CCL3*, *GDF15*, *MMP3*, *PAI-1*, *FAS*, and *SIRT1* in OA and fractured patients were determined using the 2^−ΔΔCT^ method, with normalization to the housekeeping gene β-actin as internal controls.

### 2.8. Statistical Analysis

All statistical analyses were conducted using GraphPad Prism 8 software (GraphPad Prism 8.0.1, La Jolla, CA, USA). Data from all experimental procedures are presented as mean ± standard error, and sample variance (s^2^) was calculated for each analysis. Statistical comparisons were performed using the Mann–Whitney test for data that did not meet the assumption of normality and Welch’s parametric test for data with normal distribution. Differences were considered statistically significant when *p* < 0.05.

## 3. Results

### 3.1. Clinical Profile and Muscle Histology in OA and Fractured Patients

A total of 26 subjects were enrolled in our study, divided into two groups after clinical diagnosis: 13 patients undergoing hip arthroplasty for osteoporotic fracture (fractured) and 13 patients without fragility fracture undergoing hip arthroplasty for coxarthrosis (OA). Their clinical characteristics are presented as mean ± standard error and summarized in Table 2, with the corresponding statistical analysis.

Briefly, no statistically significant differences in age and body mass index (BMI) were found between OA and fractured patients. In contrast, *T*-score values at different skeletal sites showed significant differences between the groups, with lower values in fractured patients. In addition, a statistically significant difference between the two experimental groups was also found for serum levels of 25-(OH)-Vit D, with higher values in the OA group, and parathyroid hormone (PTH), with higher values in the fractured group.

**Figure 1:**
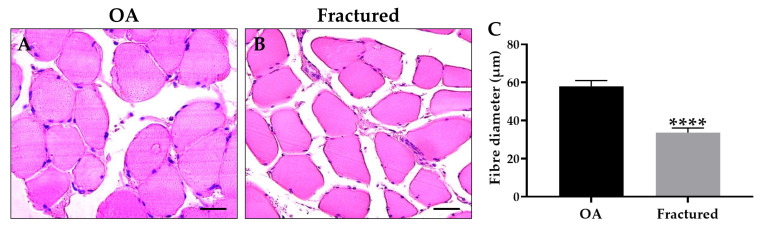
Histological and morphometric analysis of muscle tissue of osteoarthritic (OA) and fractured patients. (**A**,**B**) Hematoxylin and eosin (H&E)-stained sections of muscle tissue from patients. For 40× images, scale bar represents 50 μm. (**C**) The graph shows the mean diameter of muscle fibers measured in OA and fractured patients (**** *p* < 0.0001, s^2^ OA: 117.3, s^2^ fractured = 78.0).

Notably, histological and morphometric analysis showed a marked impairment of muscle quality in the fractured group, manifested by significant muscle atrophy, as shown in Figure 1A–C.

### 3.2. mRNA Expression of Cell Cycle Regulators and Factors Released by Aged Cells in Muscle Tissue of OA and Fractured Patients

**Figure 2:**
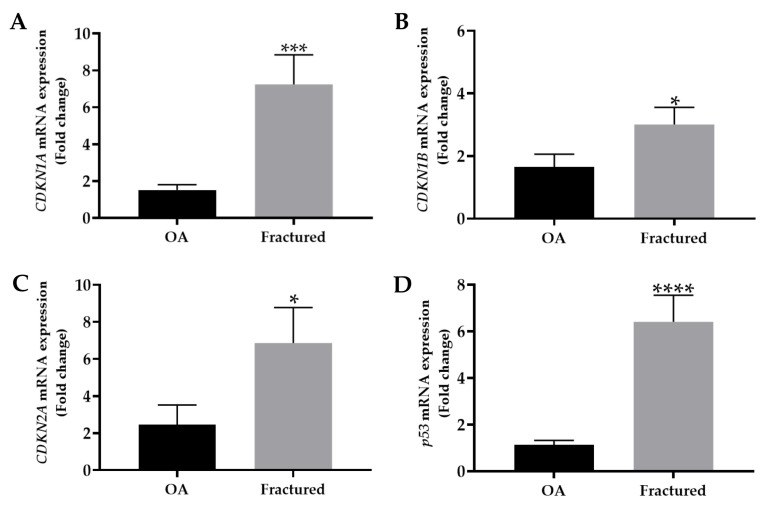
mRNA expression of senescence markers in muscle tissue from osteoarthritic (OA) and fractured patients by qRT-PCR analysis. (**A**) Mean expression levels of *CDKN1A* mRNA in the muscle tissue of OA and fractured patients (*** *p* < 0.001). (**B**) Mean expression levels of *CDKN1B* mRNA in muscle tissue of OA and fractured patients (* *p* < 0.05). (**C**) Mean expression levels of *CDKN2A* mRNA in the muscle tissue of OA and fractured patients (* *p* < 0.05). (**D**) Mean expression levels of *p53* mRNA in the muscle tissue of OA and fractured patients (**** *p* < 0.0001).

The mRNA expression levels of mainly cell cycle regulators, including *CDKN1A*, *CDKN1B*, *CDKN2A*, and *p53*, were analyzed by qRT-PCR in muscle tissue samples taken from OA and fractured patients. Importantly, the analysis revealed a significant up-regulation of these genes in the fractured group, suggesting their relevant contribution to osteoporosis-associated muscle changes (Figure 2A–D).

Table 3 summarizes the mRNA expression levels of cell cycle regulators, with the corresponding statistical analysis.

In parallel, the expression profile of investigated factors released by aged cells, such as *TNF-α*, *IL-1β*, *IL-6*, *IL-15*, *CCL2*, *CCL3*, *GDF15*, *MMP3*, *PAI-1*, and *FAS*, was examined in the muscle tissue of OA and fractured patients by qRT-PCR analysis.

**Figure 3:**
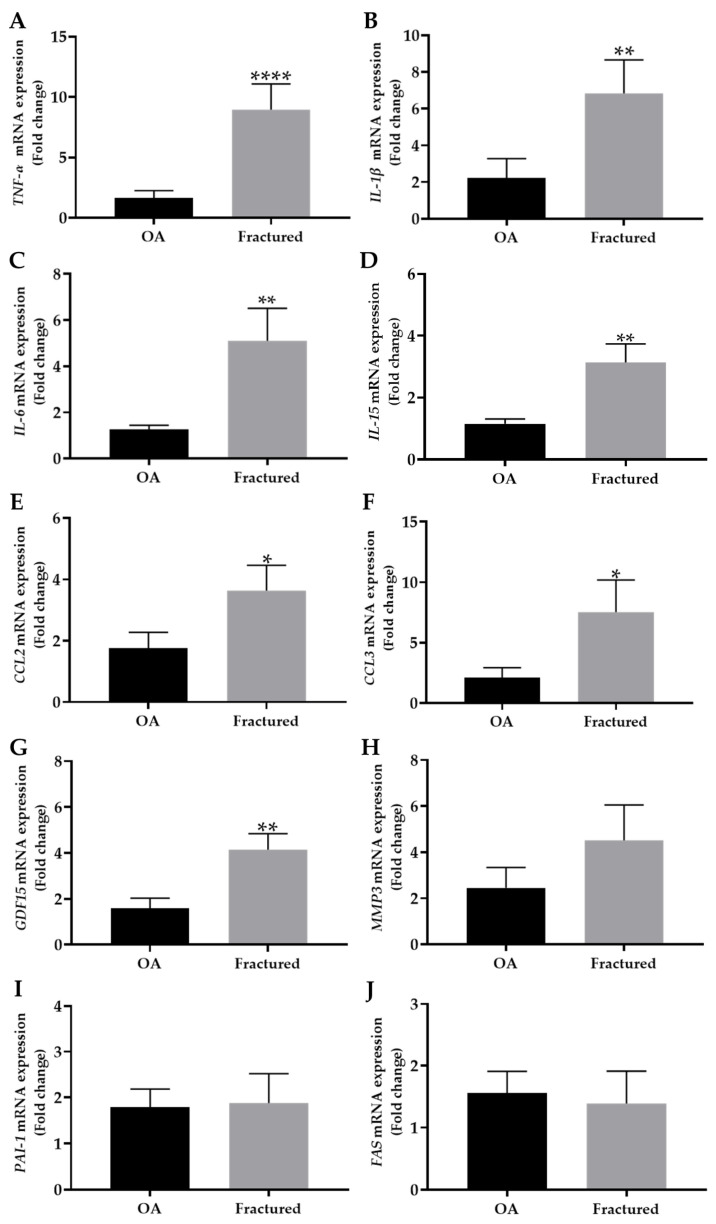
mRNA expression of senescence-associated secretory phenotype (SASP) in muscle tissue from osteoarthritic (OA) and fractured patients by qRT-PCR analysis. (**A**–**J**) mRNA expression levels of tumor necrosis factor alpha (*TNF-α*), interleukin-1 beta (*IL-1β*), interleukin-6 (*IL-6*), interleukin-15 (*IL-15*), chemokine (C-C motif) ligand 2 (*CCL2*), chemokine (C-C motif) ligand 3 (*CCL3*), growth differentiation factor 15 (*GDF15*), metallopeptidase 3 (*MMP3*), plasminogen activator inhibitor 1 (*PAI-1*), and fas cell surface death receptor (*FAS*) in the muscle tissue of OA and fractured patients. (* *p* < 0.05, ** *p* < 0.01, **** *p* < 0.0001).

Noteworthy, Figure 3A–G shows a significant increase in the expression of *TNF-α*, *IL-1β*, *IL-6*, *IL-15*, *CCL2*, *CCL3*, and *GDF15* in the fractured group compared with OA patients, suggesting a differential regulation of these markers in muscle tissue depending on the pathology considered.

In contrast, no significant differences were observed in the mRNA expression levels of *MMP3*, *PAI-1*, or *FAS*, which are known to be associated with extracellular matrix remodeling and apoptosis [28,29], between the two experimental groups (Figure 3H–J).

Table 4 summarizes the mRNA expression levels of released factor by senescent muscle cells, with the corresponding statistical analysis.

### 3.3. SIRT1 Expression in Muscle Tissue of OA and Fractured Patients

Possible variations in the SIRT1 expression pattern were investigated in the muscle tissue of OA and fractured patients, aiming to explore its involvement in the cellular aging that characterizes age-related musculoskeletal disorders.

**Figure 4:**
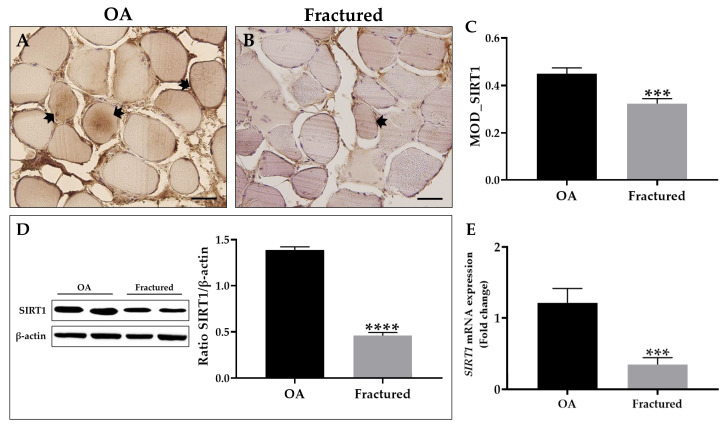
Analysis of sirtuin 1 (SIRT1) expression in muscle tissue from osteoarthritic (OA) and fractured patients. (**A**,**B**) Immunohistochemistry for SIRT1 performed on muscle tissue from OA and fractured patients. Arrows indicate representative SIRT1-positive areas. For 40× images, scale bar represents 50 μm. (**C**) Mean optical density (MOD) values for SIRT1 measured in the muscle of the OA and fractured patients (*** *p* < 0.001). (**D**) Western blotting analysis of SIRT1 performed on muscle tissue from OA and fractured patients (**** *p* < 0.0001). (**E**) qRT-PCR analysis of *SIRT1* performed on muscle tissue of OA and fractured patients (*** *p* < 0.001).

First, an immunohistochemical analysis was performed using MOD as a measurement value to quantify the expression levels of this protein, showing a significant reduction of SIRT1 in the muscle tissue of fracture patients (Figure 4A–C). These data agree with the results from Western blotting analysis, which highlighted a positive band at approximately 110 kDa, corresponding to the molecular weight of SIRT1 in the protein extracts of all tissue samples. Specifically, Figure 4D shows how SIRT1 was more expressed in the muscle tissue of OA patients respect to the fractured group. Importantly, qRT-PCR analysis confirmed these results, highlighting a significant reduction in *SIRT1* expression in the muscle tissue of fractured patients (Figure 4E).

Table 5 summarizes the expression levels of SIRT1, with the corresponding statistical analysis.

## 4. Discussion

The accumulation of aged cells compromises tissue homeostasis and promotes the progression of age-related musculoskeletal disorders, leading to the loss of muscle mass and strength [30,31]. Aged muscle cells, particularly those that overexpress p21, release factors that could act as SASPs, which promote replicative senescence in surrounding cells, inducing the arrest of cell proliferation and chronic inflammation [32,33]. This effect, known as bystander, causes thinning of muscle fibers, accelerating muscle decline associated with aging [34]. In addition, because of the mutual interaction between muscles and bones, muscle aging can promote the development and progression of osteoporosis, exacerbating musculoskeletal deterioration and promoting the onset of fragility fracture [35]. However, few studies have analyzed the expression profile of the cells cycle regulators and factors released by aged cells at the tissue level, and the available evidence mainly concerns murine models, with less focus on humans. Therefore, our pilot study aimed to investigate the expression levels of key cellular aging markers and relevant secretory factors of aging in the muscle tissue of 26 fractured and OA patients undergoing hip arthroplasty, as well as to examine the expression of SIRT1 to explore its possible role in the cellular aging processes underlying skeletal muscle atrophy. To our knowledge, this is the first evidence aimed at profiling the expression pattern potentially involved in the skeletal muscle aging of patients with or without fragility fracture.

Our results showed significant up-regulation of the cell cycle inhibitors *CDKN1A*, *CDKN1B*, and *CDKN2A*, as well as negative regulator of cell cycle *p53*, in tissue samples from fractured patients compared with the OA group, in association with reduced muscle fiber diameter and lower *T*-score values. These data agree with the study by Englund and colleagues, who previously observed how p21 overexpression induced a senescent program characterized by mitochondrial dysfunction, DNA damage, and a pronounced SASP phenotype in a mouse model, resulting in atrophy, fibrosis, and reduced muscle mass. Interestingly, these effects were accompanied by a decline in physical performance, suggesting a key role for p21 accumulation and cellular aging in muscle dysfunction associated with aging [36]. In this regard, several studies point out that the expression of cell cycle inhibitors, such as p21 and p16, varies among different cell types and tissues, each of which has a unique and characteristic SASP profile [37,38]. However, although common components have been identified in the SASP profiles of cells expressing p16 and p21, tissue-specific differences highlight the importance of single-cell studies to fully understand and characterize senescence phenomena in aging tissues. Interestingly, Fox and colleagues investigated p53 expression in the muscle of mice subjected to unilateral hindlimb immobilization, finding an association between increased p53 expression, marked muscle atrophy, and up-regulation of CDKN1A [39].

Importantly, qRT-PCR analysis showed an up-regulation of several potential SASPs factors, including *TNF-α*, *IL-1β*, *IL-6*, *IL-15*, *CCL2*, *CCL3*, and *GDF15*, in the muscle tissue of fractured patients, suggesting an association between osteoporosis and a muscle microenvironment characterized by increased inflammation and structural degradation processes. In this context, the acute inflammatory response triggered by fracture is known to involve the secretion of TNF-α, IL-1β, IL-6, and other cytokines that recruit inflammatory cells and induce angiogenesis, promoting the reparative process [40]. On the other hand, low-grade systemic inflammation, characterized by chronic expression of these inflammatory factors, is a risk factor for osteoporosis and fragility fracture [41]. In fact, our data showed increased muscle expression of these factors in fracture patients concomitantly with increased expression of negative cell cycle regulators, suggesting the crucial role of skeletal muscle cell senescence in bone fragility. These results are in agreement with those of Li and colleagues, who found a significant increase in TNF-α in the plasma of patients with sarcopenia compared to non-sarcopenic subjects [42]. Similarly, Chang et al. investigated the circulating levels of TNF-α, IL-1β, and IL-6 in sarcopenic patients, finding a significant increase compared to subjects without sarcopenia [43]. Furthermore, Parker and colleagues investigated the existence of a link between senescence and disuse muscle atrophy in a mouse model of hind limb immobilization, finding the concomitant up-regulation of IL-1β and CDKN2A [44]. Importantly, IL-6 is known to stimulate osteoclastogenesis and bone resorption, thus establishing a link between muscle senescence and bone loss [45]. In this regard, Ding et al. published the results of a meta-analysis of cross-sectional studies investigating the relationship between serum concentrations of IL-6 and the prevalence of sarcopenia in the elderly population. An inverse relationship between serum IL-6 levels and muscle mass was found, suggesting a potential role of this factor in muscle tissue impairment during aging [46]. Moreover, the secretion of chemokines, such as CCL2 and CCL3, underscores the inflammatory context associated with osteoporosis, which could further accelerate bone remodeling processes [47]. Interestingly, Kedlian et al. mapped the aging process of human intercostal muscle, detecting up-regulation of CCL2 in mononuclear muscle stem cells and of CCL3 in multinucleated myofibers, overall highlighting the participation of these factors in skeletal muscle aging [48]. In addition, Hong et al. measured mRNA levels *GDF15* in the square pronator muscle of osteoporotic women with distal wrist fracture and healthy/osteopenic women. Interestingly, muscle expression levels of *GDF15* were significantly higher in osteoporotic women, in association with increased levels of *IL-1β* and *TNF-α*, suggesting a role in the maintenance of muscle mass and function, as well as bone metabolism [49].

On the other hand, the analysis of other molecules, such as *MMP3*, *PAI-1*, and *FAS*, which are known to be involved in cellular senescence, did not show relevant differences in our samples. Nevertheless, some authors have found a relationship between MMP3, PAI-1, and FAS and skeletal muscle senescence, highlighting the need to further investigate the role of these factors in sarcopenia. Francis et al. chemically induced senescence in primary cultures of human myoblasts, finding a significant increase in *MMP3* mRNA levels [50], while Aihemaiti and colleagues observed increased muscle fiber area in middle-aged mice treated with a PAI-1 inhibitor, hypothesizing a role in age-related muscle atrophy [51]. Furthermore, Pistilli and colleagues found increased levels of TNF-α and FAS receptor mRNA in the skeletal muscle of aged rats, suggesting its involvement in the nuclear apoptosis that occurs during age-associated sarcopenia [52].

Interestingly, SIRT1 is known for its crucial role in maintaining muscle health and modulating inflammation during aging [53]. In healthy conditions, SIRT1 promotes muscle homeostasis by inhibiting pro-inflammatory nuclear factor kappa-light-chain-enhancer of activated B cells (NF-κB) signaling and supporting muscle regeneration through regulation of satellite cells [54]. On the other hand, reduced NAD levels and alterations in SIRT1 expression, which occur with aging, contribute to the decline in muscle regeneration capacity and increased chronic inflammatory state [55]. Notably, Lakhdar and colleagues analyzed vastus lateralis muscle biopsies from patients with obstructive pulmonary disease and low lean mass index, finding a trend of increasing CDKN2A/p16 in association with a significant reduction in protein levels of SIRT1 [56]. In addition, the accumulation of p21 and p27 has been observed in aged muscle cells [57], while the role of SIRT1 in their proliferation is widely recognized and well documented [58]. In accordance, our results showed increased expression of SIRT1, both at the protein and mRNA level, in the muscle tissue of OA patients compared with fractured patients, confirming its involvement in modulating the processes of muscle degeneration and inflammation, as well as maintaining cellular homeostasis. Indeed, SIRT1 is implicated in deacetylation of key transcription factors, including p53, suppressing senescence pathways and mitigating SASP-induced inflammation [59]. Several studies suggest that SIRT1 has strong anti-inflammatory effects as it can suppress the expression of inflammatory cytokines, counteracting the progression of muscle aging and sarcopenia [60]. In addition, SIRT1 activation has been shown to improve mitochondrial function and promote autophagy, processes essential for skeletal muscle health and regeneration [61]. Therefore, developing therapeutic strategies to enhance SIRT1 expression and activity could improve muscle mass and strength and mitigate the deleterious effects of factors released by cells during aging, promoting musculoskeletal health and reducing the risk of fragility fractures. In this context, the effectiveness of regular exercise, as well as caloric restriction, is known to preserve musculoskeletal health through the expression of SIRT1, suggesting the possibility of counteracting bone and muscle decline through its modulation [62,63,64].

Overall, our results show a different expression pattern of negative cell cycle regulators in the muscle of OA and fractured patients, suggesting that cellular senescence in skeletal muscle may promote bone fragility. In agreement, our results showed higher muscle expression of some important SASP factors in fractured patients compared with the OA group, confirming the crucial role of cellular senescence in muscle atrophy associated with bone fragility. Nevertheless, it is important to point out that joint pathology in OA patients could influence bone and muscle metabolism, highlighting the need to conduct further studies aimed at investigating the pattern of cellular senescence in patients without musculoskeletal pathology.

## 5. Conclusions

The interaction between bone and muscle is a central element, both structurally and biochemically, underscoring the need for a bidirectional approach for effective management of musculoskeletal diseases. Cellular aging plays a crucial role in the progression of these conditions, contributing to muscle atrophy and functional decline through the release of factors by aged cells. In this context, the development of strategies based on the use of senolytics and senomorphs, which can selectively eliminate aged cells or modulate the effects of factors released by them, represents a promising strategy to slow the pathological course and improve tissue homeostasis. Our results suggest that SIRT1 might play a role in the process of bone fragility-related muscle atrophy, as its expression is down-regulated in patients with fragility fracture. Integrating innovative drug therapies with nonpharmacological interventions, such as regular physical exercise and caloric restriction, known to promote SIRT1 activation could contribute to mitigating the progression of osteoporosis and reduce the risk of fragility fractures. However, further studies and experiments are necessary to fully understand the role of cellular aging leading to senescence under these conditions. The role of SIRT1 through its activation in skeletal muscle tissue will need to be further investigated, contributing to the development of targeted interventions that can counteract its effects on musculoskeletal health. The analysis of biopsy samples free from musculoskeletal pathologies, taken from subjects of different age groups, could provide key insights into the mechanism of cellular senescence associated with musculoskeletal disorders, promoting the development of personalized therapies based on the patient’s health condition.

## 6. Limitations of the Study

Our pilot study aimed to examine different expression patterns of senescence-driving cell cycle inhibitors, factors released by aged cells and SIRT1 in skeletal muscle under different conditions of bone integrity. Although this approach may provide a starting point for the development of personalized anti-aging therapies to reduce musculoskeletal damage, the results obtained must be considered to be preliminary. In fact, the small size of the study population, which may explain the lack of significance in the expression levels of some investigated factors released by aged cells, is due to the difficulty of enrolling elderly patients with similar characteristics without other diseases affecting musculoskeletal metabolism. Moreover, the joint pathology of OA patients, included as controls in the present study due to the absence of bone frailty, could influence musculoskeletal metabolism, suggesting the need for further studies involving subjects of different age groups, both with and without musculoskeletal disorders. However, emerging data emphasize the variability in the expression of specific factors related to muscle aging in the presence of bone fragility fractures, offering insights for further investigation.

## Figures and Tables

**Table 1:** qRT-PCR primer sequences.

Gene		Sequence (5′–3′)
*CDKN1A*	Forward	GACACCACTGGAGGGTGACT
	Reverse	CAGGTCCACATGGTCTTCCT
*CDKN1B*	Forward	ATAAGGAAGCGACCTGCAACCG
	Reverse	TTCTTGGGCGTCTGCTCCACAG
*CDKN2A*	Forward	CTTCCTGGACAGGCTGGTG
	Reverse	ATGGTTACTGCCTCTGGTGC
*p53*	Forward	TAAGCGAGCACTGCCCAACA
	Reverse	TCCTTGAGTTCCAAGGCCTC
*TNF-α*	Forward	CCTCTCTCTAATCAGCCCTCTG
	Reverse	GAGGACCTGGGAGTAGATGAG
*IL-1β*	Forward	ATGATGGCTTATTACAGTGGCAA
	Reverse	GTCGGAGATTCGTAGCTGGA
*IL-6*	Forward	GGTACATCCTCGACGGCATCT
	Reverse	GTGCCTCTTTGCTGCTTTCAC
*IL-15*	Forward	CATGGTATTGGGAACCATAGATTTG
	Reverse	CATTCACCCAGTTGGCTTCTG
*CCL2*	Forward	AGAATCACCAGCAGCAAGTGTC
	Reverse	TCCTGAACCCACTTCTGCTTGG
*CCL3*	Forward	AGCTGACTACTTTGAGACGAGCA
	Reverse	CGGCTTCGCTTGGTTAGGA
*GDF15*	Forward	CACCCTCAGAGTTGCACTCC
	Reverse	GCCTGGTTAGCAGGTCCTC
*MMP3*	Forward	CACTCACAGACCTGACTCGGTT
	Reverse	AAGCAGGATCACAGTTGGCTGG
*PAI-1*	Forward	CTCATCAGCCACTGGAAAGGCA
	Reverse	GACTCGTGAAGTCAGCCTGAAAC
*FAS*	Forward	TGAAGGACATGGCTTAGAAGTG
	Reverse	GGTGCAAGGGTCACAGTGTT
*SIRT1*	Forward	TAGACACGCTGGAACAGGTTGC
	Reverse	CTCCTCGTACAGCTTCACAGTC
*β-ACTIN*	Forward	ACTCCATGCCCAGGAAGGAA
	Reverse	GAGATGGCCACGGCTGCTT

qRT-PCR: real-time quantitative polymerase chain reaction; *CDKN1A*: cyclin-dependent kinase inhibitor 1A; *CDKN1B*: cyclin-dependent kinase inhibitor 1B; *CDKN2A*: cyclin-dependent kinase inhibitor 2A; *TNF-α*: tumor necrosis factor α; *IL-1β*: interleukin 1β; *IL-6*: interleukin-6; *IL-15*: interleukin-15; *CCL2*: chemokine (C-C motif) ligand 2; *CCL3*: chemokine (C-C motif) ligand 3; *GDF15*: growth differentiation factor 15; *MMP3*: matrix metallopeptidase 3; *PAI-1*: plasminogen activator inhibitor 1; *FAS*: fas cell surface death receptor; *SIRT1*: sirtuin 1.

**Table 2:** Clinical parameters of patients.

Parameters	OA (*n* = 13)	Fractured (*n* = 13)	*p*-Value	Variance (s^2^)
Age (years)	75.1 ± 7.0	74.3 ± 7.9	*p* = 0.7949	OA: 49.1 Fractured: 62.2
BMI (Kg/m^2^)	27.2 ± 5.1	26.8 ± 3.4	*p* = 0.7954	OA: 26.0 Fractured: 11.6
*T*-score (L1–L4)	1.0 ± 0.8	-2.6 ± 0.6	**** *p* < 0.0001	OA: 0.7 Fractured: 0.4
*T*-score (femoral neck)	0.4 ± 1.2	-2.5 ± 0.6	**** *p* < 0.0001	OA: 1.5 Fractured: 0.3
*T*-score (total femur)	0.7 ± 1.5	-2.8± 0.6	**** *p* < 0.0001	OA: 2.3 Fractured: 0.4
25-(OH)-Vit D (ng/mL)	20.1 ± 9.5	13.4 ± 6.0	* *p* < 0.05	OA: 89.5 Fractured: 36.6
PTH (pg/mL)	72.2 ± 21.6	111.8 ± 11.2	**** *p* < 0.0001	OA: 465.1 Fractured: 125.7

OA: patients undergoing hip arthroplasty for coxarthrosis; BMI: body mass index; PTH: parathyroid hormone.

**Table 3:** mRNA expression levels of *CDKN1A*, *CDKN1B*, *CDKN2A*, and *p53*.

Gene	OA (*n* = 13)	Fractured (*n* = 13)	*p*-Value	Variance (s^2^)
*CDKN1A*	1.5 ± 0.3	7.2 ± 1.6	*** *p* < 0.001	OA: 1.1 Fractured: 33.6
*CDKN1B*	1.6 ± 0.4	3.0 ± 0.5	* *p* < 0.05	OA: 2.2 Fractured: 3.9
*CDKN2A*	2.4 ± 1.1	6.9 ± 1.9	* *p* < 0.05	OA: 15.0 Fractured: 47.6
*p53*	1.1 ± 0.2	6.4 ± 1.1	**** *p* < 0.0001	OA: 0.5 Fractured: 17.0

OA: patients undergoing hip arthroplasty for coxarthrosis; *CDKN1A*: cyclin-dependent kinase inhibitor 1A; *CDKN1B*: cyclin-dependent kinase inhibitor 1B; *CDKN2A*: cyclin-dependent kinase inhibitor 2A.

**Table 4:** mRNA expression levels of *TNF-α*, *IL-1β*, *IL-6*, *IL-15*, *CCL2*, *CCL3*, *GDF15*, *MMP3*, *PAI-1*, and *FAS*.

Gene	OA (*n* = 13)	Fractured (*n* = 13)	*p*-Value	Variance (s^2^)
*TNF-α*	1.7 ± 0.6	9.0 ± 2.1	**** *p* < 0.0001	OA: 5.2 Fractured: 59.0
*IL-1β*	2.2 ± 1.0	6.8 ± 1.8	** *p* < 0.01	OA: 13.1 Fractured: 40.1
*IL-6*	1.2 ± 0.2	5.1 ± 1.4	** *p* < 0.01	OA: 0.5 Fractured: 25.6
*IL-15*	1.1 ± 0.1	3.1 ± 0.6	** *p* < 0.01	OA: 0.3 Fractured: 4.6
*CCL2*	1.8 ± 0.5	3.6 ± 0.8	* *p* < 0.05	OA: 3.1 Fractured: 8.0
*CCL3*	2.1 ± 0.8	7.5 ± 2.6	* *p* < 0.05	OA: 7.0 Fractured: 70.3
*GDF15*	1.6 ± 0.4	4.1 ± 0.7	** *p* < 0.01	OA: 2.5 Fractured: 6.3
*MMP3*	2.4 ± 0.9	4.5 ± 1.5	*p* = 0.6139	OA: 10.1 Fractured: 30.5
*PAI-1*	1.8 ± 0.4	1.9 ± 0.6	*p* = 0.8010	OA: 2.0 Fractured: 5.3
*FAS*	1.5 ± 0.3	1.4 ± 0.5	*p* = 0.3358	OA: 1.6 Fractured: 3.5

OA: patients undergoing hip arthroplasty for coxarthrosis; *TNF-α*: tumor necrosis factor α; *IL-1β*: interleukin 1β; *IL-6*: interleukin-6; *IL-15*: interleukin-15; *CCL2*: chemokine (C-C motif) ligand 2; *CCL3*: chemokine (C-C motif) ligand 3; *GDF15*: growth differentiation factor 15; *MMP3*: matrix metallopeptidase 3; *PAI-1*: plasminogen activator inhibitor 1; *FAS*: fas cell surface death receptor.

**Table 5:** Evaluation of SIRT1 expression by immunohistochemistry, Western blotting, and qRT-PCR.

SIRT1 Expression	OA (*n* = 13)	Fractured (*n* = 13)	*p*-Value	Variance (s^2^)
Immunohistochemistry (MOD value)	0.45 ± 0.02	0.32 ± 0.02	*** *p* < 0.001	OA: 0.01 Fractured: 0.01
Western Blotting (protein levels)	1.39 ± 0.04	0.46 ± 0.05	**** *p* < 0.0001	OA: 0.01 Fractured: 0.01
qRT-PCR (mRNA levels)	1.2 ± 0.2	0.3 ± 0.1	*** *p* < 0.001	OA: 0.5 Fractured: 0.1

OA: patients undergoing hip arthroplasty for coxarthrosis; SIRT1: sirtuin 1; qRT-PCR: real-time quantitative polymerase chain reaction; MOD: mean optical density.

## Data Availability

The original contributions presented in the study are included in the article/Supplementary Material. Further inquiries can be directed to the corresponding author.

## Supplementary Materials

The following supporting information can be downloaded at: https://www.mdpi.com/article/10.3390/biomedicines13061350/s1, Figure S1: Analysis of sirtuin 1 (SIRT1) expression in muscle tissue of osteoarthritic (OA) and fractured patients.

## Abbreviations

The following abbreviations are used in this manuscript:

SIRT1	Sirtuin 1	
OA	Patients undergoing hip arthroplasty for coxarthrosis	
CDKN1A	Cyclin-dependent kinase inhibitor 1A	
CDKN1B	Cyclin-dependent kinase inhibitor 1B	
CDKN2A	Cyclin-dependent kinase inhibitor 2A	
TNF-α	Tumor necrosis factor alpha	
IL-1β	Interleukin-1 beta	
IL-6	Interleukin-6	
IL-15	Interleukin-15	
CCL2	Chemokine (C-C motif) ligand 2	
CCL3	Chemokine (C-C motif) ligand 3	
GDF15	Growth differentiation factor 15	
MMP3	Matrix metallopeptidase 3	
PAI-1	Plasminogen activator inhibitor 1	
FAS	Fas cell surface death receptor	
SASP	Senescence-associated secretory phenotype	
NAD	Nicotinamide adenine dinucleotide	
BMD	Bone mineral density	
DXA	Dual-energy X-ray absorptiometry	
CET	Territorial ethics committee	
H&E	Hematoxylin and eosin	
EDTA	Ethylenediaminetetraacetic acid	
HRP	Horseradish peroxidase	
DAB	3,3’-diaminobenzidine	
MOD	Mean optical density	
qRT-PCR	Real-time quantitative polymerase chain reaction	
BMI	Body mass index	
PTH	Parathyroid hormone	
NF-κB	Kappa-light-chain-enhancer of activated B cells	
