# Supplementary material for: Altered Expression of Cell Cycle Regulators and Factors Released by Aged Cells in Skeletal Muscle of Patients with Bone Fragility: A Pilot Study on the Potential Role of SIRT1 in Muscle Atrophy
Source: Biomedicines. 2025 May 31;13(6):1350. doi: 10.3390/biomedicines13061350 (PMC12190532; doi:10.3390/biomedicines13061350)
Supplement: Supplementary file 1 [file biomedicines-13-01350-s001.zip › biomedicines-3620215-supplementary.pdf]

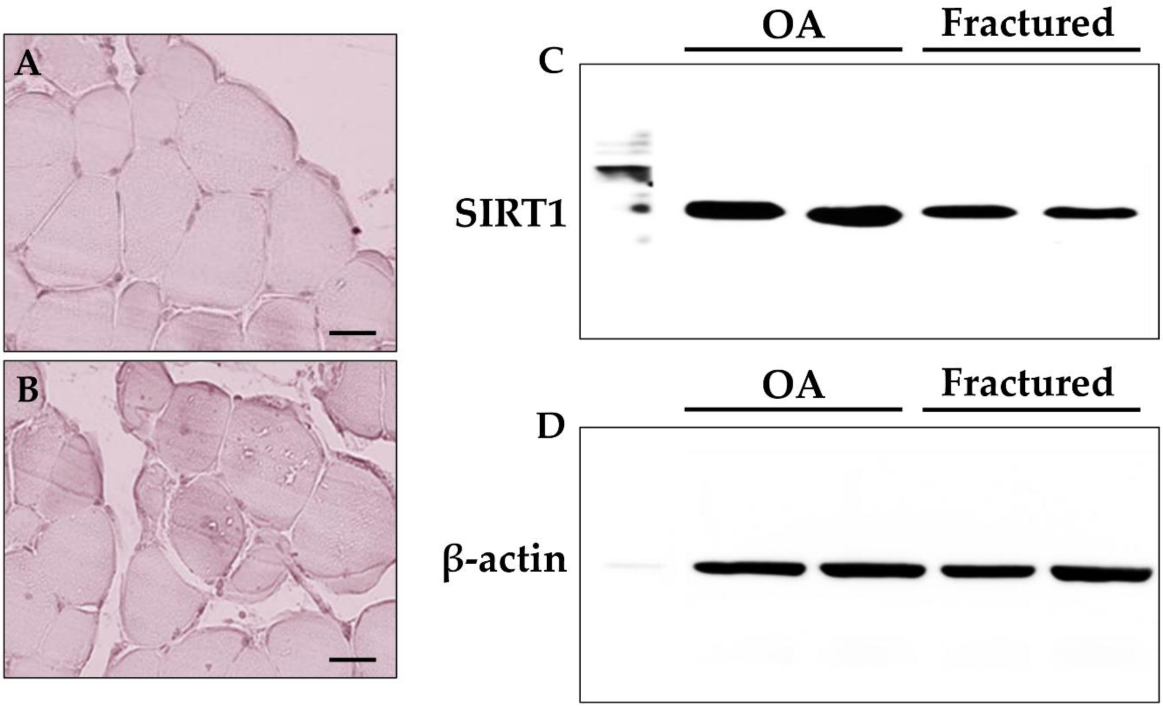

Figure S1. Analysis of sirtuin 1 (SIRT1) expression in muscle tissue of osteoarthritic (OA) and fractured patients. (A,B) Immunohistochemistry: (A) SIRT1-negative control in the muscle tissue from OA patients. (B) SIRT1-negative control in the muscle tissue from fractured patients. For 40× images, scale bar represents 50 µm. (C,D) Original western blotting images: (C) SIRT1 expression (molecular weight 110 kDa) in muscle tissue from OA and fractured patients. (D) β-actin expression (molecular weight 42 kDa) in muscle tissue from OA and fractured patients.
